# Designable polypyrrole pattern in hydrogel achieved by photo‐controllable concentration of Fe^3+^ initiator

**DOI:** 10.1002/smo.20240015

**Published:** 2024-06-27

**Authors:** Xinyu Zhao, Huidong Xu, Zhao‐Tie Liu, Guo Li, Jinqiang Jiang, Zhong‐Wen Liu

**Affiliations:** ^1^ Key Laboratory of Syngas Conversion of Shaanxi Province School of Chemistry and Chemical Engineering Shaanxi Normal University Xi'an Shaanxi Province China

**Keywords:** conductive pattern, Fe^3+^, hydrogels, photothermal effect, polypyrrole

## Abstract

Conductive polymer hydrogels (CPHs) are promising in cutting‐edge applications including bioelectronics and tissue engineering. However, the precise regulation of the spatial distribution of the conductive polymer (CP) in the hydrogel network is still an issue for designing a smart material. Herein, we propose a facile method for preparing CPH‐based smart materials by controlling the distribution of Fe^3+^ initiator with UV light irradiation. Thus, designable polypyrrole (PPy) conductive patterns in the polyvinyl alcohol/sodium alginate (PVA/SA) semi‐interpenetrating hydrogel network are demonstrated by controlling the concentration of Fe^3+^ ions coordinated with carboxylate groups. Depending on the irradiation time, the reduction of Fe^3+^ to Fe^2+^ occurs in different extents. As a result, the controllable polymerization of pyrrole only initiated by Fe^3+^ is achieved to form desirable CPH patterns, which are confirmed by the characterization results of Fourier transform infrared spectrometry, X‐ray photoelectron spectroscopy, and scanning electron microscopy. Moreover, the developed hydrogel with PPy patterns is illustrated for the application in smart conductive circuit and information encryption. The simple procedure and the controllable conductive patterning of the proposed method make it a promising route in developing smart hydrogel materials, which can be extended to other Fe^3+^ initiated CP patterns.

## Graphical abstract

Polypyrrole patterns are created in polyvinyl alcohol/sodium alginate physical hydrogels. This is realized by spatially regulating the concentration of Fe^3+^ ions acting as the initiator of pyrrole polymerization via controlling the time of UV light irradiation. The developed hydrogels with polypyrrole patterns show designable electric conductivity and photothermal effect, which can be applied in fields such as smart circuit and information encryption.

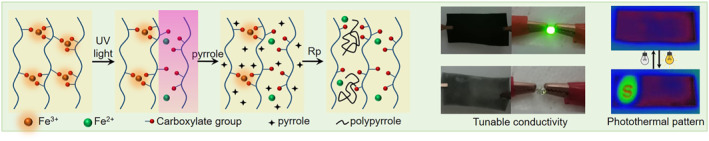

## 1 INTRODUCTION

Conductive polymer hydrogels (CPHs) have gained a wide scope of research interests due to their metal‐like high conductivity and flexible mechanical properties, which are promising in various applications including bioelectronics, tissue engineering, and artificial skin.[^1^, ^2^, ^3^, ^4^, ^5^] The typical structure of CPHs is a three‐dimensional crosslinked polymer network incorporated with conductive ingredients and a large amount of water. The commonly adopted conductive ingredients include metals in the form of ions such as Na^+^ and Al^3+^ or nanomaterials including 1D rods and 2D flakes, carbon nanomaterials (carbon nanotubes, graphene, etc.), and conductive polymers (CPs), especially polyaniline and polypyrrole (PPy).[^6^, ^7^, ^8^, ^9^, ^10^] Among these materials, CPs are advantageous in their light‐weight, biocompatibility, and superior photo‐ and electrothermal properties. Current research on CP‐containing hydrogels is focused on improving conductivity, enhancing mechanical properties, or combining with other functionalities such as self‐healing or stimuli‐driven shape‐changing behaviors. However, in most cases, the distribution of CPs is uncontrollable in hydrogel networks. This limitation greatly hinders the application of CP‐containing CPHs, especially in some cutting‐edge fields including miniaturization and integration of soft electronics and the development of implantable devices for site‐specific signal detection.[^11^, ^12^]

Various routes to develop CP patterns on different substrates have been explored, and the most notable techniques are soft lithography, electron beam lithography, photolithography, and nanoimprinting.[^13^, ^14^, ^15^, ^16^, ^17^, ^18^] However, the availability of these techniques to pattern CPs in soft and hydrophilic hydrogel networks is still questionable. Common procedures to prepare CP‐containing HAs include inducing the gelation of aqueous mixtures of polymers and CPs, post‐polymerization of corresponding monomers in hydrogels, and direct crosslinking of CPs.[^2^, ^19^] These procedures lack spatial controllability and therefore the developed HAs exhibit homogeneous CP distributions. Only very recently, several HAs with controllable CP distributions have been reported in the literature. Li and coworkers injected and polymerized pyrrole aqueous solutions in the microchannels of a polydimethylsiloxane template to develop conductive hydrogel patterns.^[20]^ Levkin et al. prepared a template with spatially differentiated surface hydrophilicity to direct the position of the monomer solution before being polymerized.^[21]^ Bao et al. reported a patterned poly(3,4‐ethylenedioxythiophene):polystyrene sulfonate (PEDOT:PSS) hydrogel prepared by electrostatically‐induced gelation of PEDOT:PSS electrolyte solution with the presence of a patterned Cu layer under external electronic field.^[22]^ Although these template‐directed polymerization methods are convenient and extendable, the intricacy of the resulting pattern determined by the template is usually low. This can be a severe drawback for fabricating micro‐sized hydrogel electronics. Wang et al. developed a PPy‐patterned hydrogel by UV light‐induced breakage of covalent bonds in bovine serum albumin chains in a PPy‐containing hydrogel film. Although micrometer‐sized patterns are created by spatiotemporal control of UV light irradiation, the existence and photo‐degradation of bovine serum albumin compromises the adaptability of this method to other hydrogel systems.^[23]^ Therefore, developing a convenient and extendable strategy for fabricating HAs with high‐precision CP patterns is of great importance and still challenging.

**FIGURE 1:**
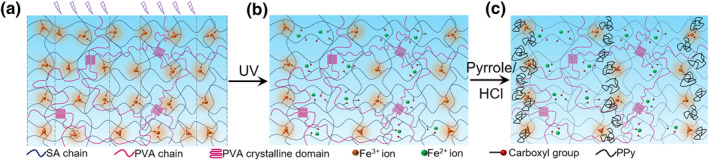
Schematic illustration of the photo‐controlled creation of the PPy pattern in PVA/SA hydrogel. (a): As‐prepared PVA/SA hydrogel; (b): introducing Fe^3+^ ions and spatially exposing it to UV light; (c) introducing pyrrole to form PPy pattern. PPy, polypyrrole; PVA, polyvinyl alcohol; SA, sodium alginate.

In this work, we report a strategy to address the thus mentioned challenge. This strategy is realized by spatially controlling the concentration of Fe^3+^ ions acting as the initiator of pyrrole polymerization. As demonstrated in Figure 1, we select physically crosslinked polyvinyl alcohol/sodium alginate (PVA/SA) as a model hydrogel. As reported in the literatures, this hydrogel is prepared by dissolving PVA and SA in water and successive freeze‐drying, leading to the crystallization of PVA and the formation of physical crosslinking structure (Figure 1a).[^24^, ^25^, ^26^] Here PVA is selected because it renders the hydrogel good geometrical stability and mechanical properties, and SA is used for providing carboxylate groups as binding sites. Fe^3+^ ions are introduced by an immersion step to coordinate with carboxylate groups, which can be photo‐reduced into Fe^2+^ ions upon UV light irradiation (Figure 1b).[^27^, ^28^, ^29^] Since Fe^3+^ can initiate the polymerization of pyrrole whereas Fe^2+^ ions cannot, the amount of polymerized PPy in the hydrogel network can be spatially regulated by controlling the concentration of Fe^3+^ ion (Figure 1c).[^30^, ^31^] The results show that PPy patterns can be conveniently created by simply controlling the UV light irradiation time, and the precision of the patterns can be sized down to micrometer scale. We also demonstrate the potential of the developed hydrogels with designed PPy patterns in applications including smart circuits and information encryption.

## 2 RESULTS AND DISCUSSION

**FIGURE 2:**
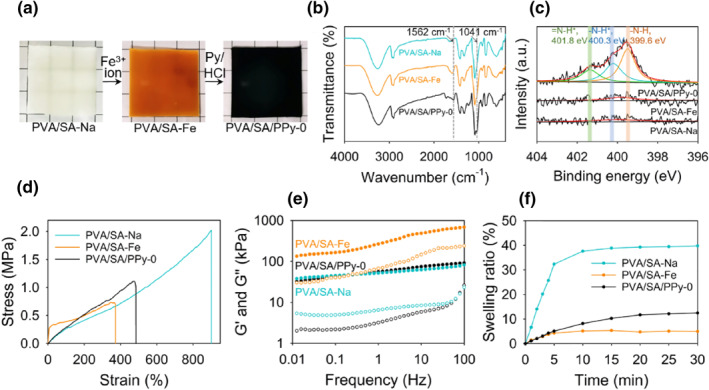
(a) Photos showing the appearance of a hydrogel sample during pyrrole polymerization. (b) FTIR spectra, (c) XPS spectra, (d) stress‐strain curves, (e) storage and loss moduli, and (f) swelling behaviors of PVA/SA‐Na, PVA/SA‐Fe, and PVA/SA/PPy‐0. FTIR, Fourier transform infrared spectra; PVA, polyvinyl alcohol; SA, sodium alginate; XPS; X‐ray photoelectron spectroscopy.

The procedure to polymerize pyrrole in‐situ in a PVA/SA hydrogel network is first demonstrated. Figure 2a shows the digital photos of a PVA/SA hydrogel sample at different states during PPy formation. The white and semi‐transparent appearance of the as‐prepared PVA/SA hydrogel sample (denoted as PVA/SA‐Na) is due to the existence of the PVA crystalline domains formed during the freezing‐thawing step. After the introduction of Fe^3+^ ions (denoted as PVA/SA‐Fe), the color of the hydrogel changes from white to orange. Further immersed in acidic pyrrole solution the sample turns into black, indicating the formation of PPy. In the following context, hydrogel samples containing PPy are labeled as PVA/SA/PPy‐x, where x indicates the irradiation time. For example, the unirradiated sample in Figure 2a is PVA/SA/PPy‐0. Fourier transform infrared spectrometry (FT‐IR) and X‐ray photoelectron spectroscopy (XPS) tests were conducted to certify the successful formation of PPy in the hydrogel. The effectiveness of UV light‐induced reduction of Fe^3+^ to Fe^2+^ is evidenced by UV‐Vis spectra, which shows gradual decrease in absorption intensity with irradiation time (Figure S1).[^27^, ^32^] Figure 2b shows the FT‐IR spectra of the three samples (PVA/SA‐Na, PVA/SA‐Fe, and PVA/SA/PPy‐0). The characteristic peaks located at 1562 and 1041 cm^−1^ in the spectrum of PVA/SA/PPy‐0 are, respectively, assigned to the C=C stretching vibration and N‐H in‐plane deformation of the pyrrole rings. However, these two peaks cannot be observed in the spectra of PVA/SA‐Na and PVA/SA‐Fe. The XPS spectra (N1 s) of these three samples are given in Figure 2c. Three characteristic peaks at 401.8, 400.3 and 399.6 eV assigned to = N−H^+^, −N−H^+^, and −N−H groups from PPy can only be found in the spectrum of PVA/SA/PPy‐0. These results clearly indicate that the polymerization of pyrrole can be successfully initiated by the Fe^3+^ ions coordinated with the carboxylate groups.

The influence of PPy formation on the hydrogel properties is next investigated. Figure 2d shows the stress‐strain curves of the three samples. The PVA/SA‐Na sample exhibits a superior toughness, as the strain at break can be as high as 901% and the tensile strength is 2.02 MPa. The introduction of Fe^3+^ ion makes the hydrogel much more brittle, as the strain at break of PVA/SA‐Fe decreases to 372%, accompanied by the simultaneous deterioration of tensile strength (decreased to 0.70 MPa). This is possibly due to the formation of excessive Fe^3+^‐carboxylate coordinates that sacrifice network elasticity. The PVA/SA/PPy‐0 sample shows better toughness than PVA/SA‐Fe, as the values of strain at break and tensile strength for PVA/SA/PPy‐0 are 479% and 1.11 MPa, respectively. It indicates that the formed PPy has a strengthening effect on the PVA/SA network. Besides elongation, the samples can also tolerate other types of deformation such as twisting and knotting without generating observable dehiscence (Figure S2). The rheological properties of the three hydrogel samples are given in Figure 2e. The storage moduli of the three samples are about 1 order of magnitude higher than the loss moduli, and the values of storage moduli of the three samples exhibit slight changes with the variation of frequency. These results indicate that the physical crosslinking formed by PVA chains is robust to hold the network as a solid. The swelling behaviors of the three samples can be found in Figure 2f. The PVA/SA‐Na sample shows a prominent swelling behavior whereas PVA/SA‐Fe and PVA/SA‐PPy‐0 are only modestly swelled within the investigated 60 min. The difference in swelling behavior is mainly due to the formation and photo‐dissociation of Fe^3+^‐carboxylate coordinates that changes the network density. From the above results, it can be concluded that the introduced Fe^3+^ ions can coordinate with carboxylate groups before successfully initiating the polymerization of PPy in‐situ in the hydrogel network. The formation and photo‐dissociation of Fe^3+^‐carboxylate coordinates can regulate hydrogel properties in a wide range, and the formed PPy can toughen the hydrogel network without altering other properties prominently. We also surveyed the influences of freezing time in freezing/thawing treatment on hydrogel properties including crystallinity, swelling behavior, and mechanical properties. The results given in Figures S3‐S6 indicate that a longer freezing time leads to higher crystallinity, lower water content and better mechanical performances. The reason lies in that a longer retention time at temperature lower than the freezing point of water leads to more sufficient phase separation of water and PVA. This favors the generation of PVA crystalline domains acting as crosslinking sites, which can toughen and make the network denser and stronger to resist volume change and stress‐induced deformation.

**FIGURE 3:**
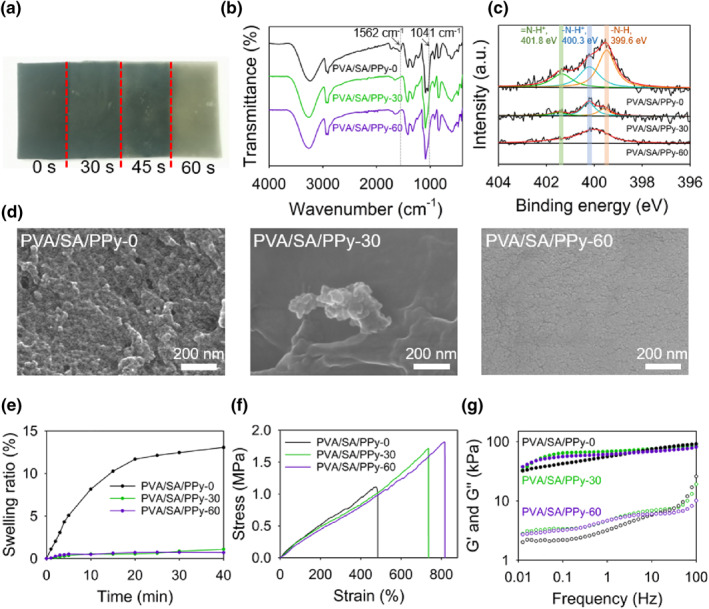
(a) An image showing a sample with developed PPy pattern. (b) FTIR spectra, (c) XPS spectra (d) SEM images, (e) swelling behaviors, (f) stress‐strain curves, and (g) storage and loss moduli of PVA/SA/PPy‐0, PVA/SA/PPy‐30, and PVA/SA/PPy‐60. FTIR, Fourier transform infrared spectra; PPy, polypyrrole; PVA, polyvinyl alcohol; SA, sodium alginate; SEM, scanning electron microscopy; XPS; X‐ray photoelectron spectroscopy.

We next show that the polymerization of pyrrole can be hindered by simply controlling the UV light irradiation time. This is realized by the fact that the photo‐reduced Fe^2+^ ions are incapable of initiating the polymerization of pyrrole. This is implemented by spatially irradiating PVA/SA‐Fe samples for different time before immersing them in acidic pyrrole solution. An example is presented in the photo given in Figure 3a, in which the four square regions reside parallelly along the length direction of the hydrogel sample. From left to right, the irradiation times for the four regions are 0,30,45 and 60 s, respectively. It can be observed that regions with less irradiation times exhibit deeper dark colors, indicating a higher concentration of PPy generated in these regions. The controllability of UV light irradiation time on the pyrrole polymerization is also verified by FT‐IR and XPS. Figure 3b shows FT‐IR spectra of PVA/SA/PPy‐0, PVA/SA/PPy‐30, and PVA/SA/PPy‐60. The intensities of the characteristic peaks located at 1562 and 1041 cm^−1^ assigned to the deformation of pyrrole rings simultaneously decrease in the spectra of samples with longer irradiation times. Similar results can be observed in the N1 s XPS spectra given in Figure 3c, as all the characteristic peaks at 401.8, 400.3, and 399.6 eV show a decreasing tendency of intensity for samples being irradiated with prolonged times. These results suggest that a longer irradiation time can effectively prevent the polymerization of pyrrole, leading to a lower concentration of PPy. The scanning electron microscopy (SEM) images given in Figure 3d reveal the microscopic cross‐section morphologies of the samples with different irradiation times. The PVA/SA/PPy‐0 sample shows a rather rough morphology with PPy particles aggregated on the hydrogel surface. On the contrary, only isolated PPy aggregates can be observed on the surface of PVA/SAPPy‐30, and for PVA/SAPPy‐60 it exhibits a rather smooth morphology with no observable PPy aggregate on the surface. These results clearly suggest the difference in PPy concentration of the investigated three samples. We further investigate the influence of PPy content on hydrogel properties. Figure 3e shows the swelling behaviors of PVA/SAPPy‐0, PVA/SAPPy‐30, and PVA/SAPPy‐60. It is interesting to see that only PVA/SAPPy‐0 shows a noticeable swelling behavior, as its fractional weight increase is 13.4% after 40 min of swelling. For PVA/SAPPy‐30 and PVA/SAPPy‐60, the weight increase is only 1.0% and 0.7%, respectively. The notable decrease of swelling ability of the hydrogel may originate from the decarboxylation of SA chains during photo‐reduction of Fe^3+^ ions, which prominently deteriorate the network hydrophilicity. The lowered water content in the hydrogel network leads to better mechanical properties, as the tress‐strain curves of the three samples given in Figure 3f, samples with longer irradiation times exhibit larger tensile strength and simultaneous higher tensile strains. The results of the rheological tests are presented in Figure 3g. The values of G′ for the three samples are quite approaching, which are one order of magnitude higher than that of G′′ and change mildly with frequency. This indicates that the UV light irradiation and PPy formation have very limited effects on the network solidity, and the formation of PPy aggregates can strengthen the hydrogel network to exhibit better mechanical properties.

**FIGURE 4:**
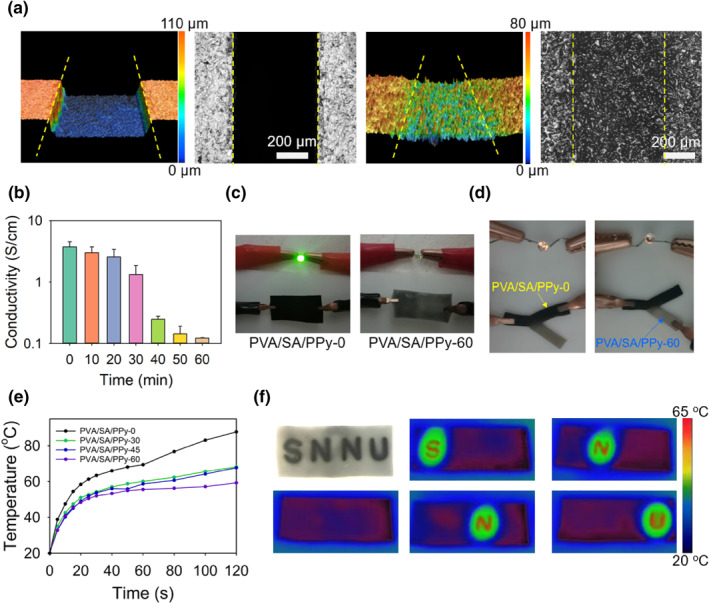
(a) CLSM and SEM images showing the hollowed pattern of the mold (left) and the created PPy pattern (right) in hydrogel. (b) The relationship between irradiation time and the electric conductivity of the hydrogel. (c) and (d) photos showing the difference in brightness of the bulb connected with two samples in different circuits. (e) Temperature rising behaviors of different samples under NIR light exposure (300 mW). (f) Digital and infrared thermal images showing a hydrogel sample with a PPy pattern composed of four letters. These letters can be read in the dark by exposing the sample to NIR light. CLSM, confocal laser scaanning microscopy; NIR, near infrared; PPy, polypyrrole; SEM, scanning electron microscopy.

The proposed method can create PPy patterns with high precisions. As demonstrated in Figure 4a, the confocal laser scanning microscopy and SEM images of a home‐made mold and the corresponding PPy pattern formed in the hydrogel are presented. The two images on the left clearly show that the mold possesses separated strip‐like hollow regions. The created PPy pattern (the two images on the right) by using this mold as photo‐mask corresponds well with the mold, as the strip‐shape of the hollow region is well kept, and the width of the region is also 500 μm (the same as the mold). The potential applications of the developed hydrogels with PPy patterns are next surveyed. Since PPy is widely applied because of its superior electric conductivity and photothermal convertibility, the spatial regulation of these two functionalities by controlling PPy concentration is demonstrated. Figure 4b shows the relationship between hydrogel conductivity and the UV light irradiation time during preparation. It can be seen that the conductivity of the unirradiated sample is as high as 3.82 S/cm, while after 60 min irradiation the conductivity sharply decreases to 0.12 S/cm. This prominent change in conductivity is also reflected by applying the hydrogels as conductive wires. As shown in the images in Figure 4c, the unirradiated sample PVA/SA/PPy‐0 was incorporated into an electric circuit connected with a battery group and a bulb. The bulb shines brightly when the circuit is connected. In contrast, when PVA/SA/PPy‐0 is replaced by PVA/SA/PPy‐60, the brightness of the bulb can hardly be observed. The sharp contract in the hydrogel conductivity can be utilized to design smart hydrogel circuits. As the sample with a Y‐shaped PPy pattern demonstrated in Figure 4d, by selecting the positions to connect into the circuit, the bulb shows a significant difference in brightness. It indicates that the hydrogels with PPy patterns may be promising in acting as smart electric switches. On another front, we show that the PPy pattern can be applied to regulate the near infrared (NIR) light‐induced temperature rise in the hydrogel. The difference in the photothermal effect of hydrogel samples with different PPy concentrations was first surveyed. The results presented in Figure 4e clearly indicate that the temperature of samples with different PPy concentrations rapidly rises with time under NIR light (300 mW). However, samples with higher PPy concentrations exhibit higher temperatures, as after 120 s of irradiation, the achieved temperatures of PVA/SA/PPy‐0, PVA/SA/PPy‐30, PVA/SA/PPy‐45, and PVA/SA/PPy‐60 are 87.7, 68.2, 67.7, and 59.3°C, respectively. This can be applied in the field of information encryption. As an example of demonstration, a hydrogel with a PPy pattern composed of four letters “S,” “N,” “N,” and “U,” which is the abbreviation of the authors' affiliation, was prepared (Figure 4f). While the four letters can be easily seen in the daytime, they can hardly be noticed in the dark. However, these letters can be read by using an infrared thermal camera, as when irradiating the hydrogel from left to right, the four letters can be, respectively, recorded by the camera.

## 3 CONCLUSION

This study proposes a novel method to create PPy patterns in hydrogels. The core idea of the method is spatial regulation of the Fe^3+^ concentration in hydrogel by controlling UV light irradiation. Since Fe^3+^ ions can initiate polymerization of pyrrole while Fe^2+^ ions cannot, the concentrations of polymerized PPy can be spatially differentiated in different regions of the hydrogels determined by the Fe^3+^ ion concentration. The created patterns can be delicate with micrometer‐level precisions, and the regions with different PPy concentrations are quite different in electric conductivity and photo‐thermal effect. Therefore, the developed hydrogels can be promisingly applied in fields including the demonstrated smart circuits and information encryption. This method is convenient and can be generally extended to develop patterns of different CPs in various hydrogel systems.

## 4 EXPERIMENTAL SECTION/METHODS

### 4.1 Materials

PVA (1799) was purchased from Chengdu Aike Reagent Co., Ltd. (China). SA was purchased from Shanghai Aladdin Biochemical Technology Co., Ltd. Pyrrole and ferric chloride hexahydrate were purchased from Beijing InnoChem Science & Technology Co., Ltd. Hydrochloric acid was purchased from Sinopharm Chemical Reagent Co., Ltd.

### 4.2 Preparation of PVA/SA hydrogel

PVA (15 wt%) was dissolved in deionized water (79 wt%) in a round‐bottom flask and heated at 95°C for 0.5 h under stirring to obtain a homogeneous solution. SA was then added and evenly mixed with PVA in solution by vigorous stirring using a metal stick at 95°C for 0.5 h. The obtained homogeneous mixture was transferred into homemade glass molds sealed using silicone rubbers. The molds have Sandwich structures, and the mixture was filled in the central square space engraved in the silicone rubber sheets. The mixtures in the molds were further treated by a freeze‐thaw cycle with 12 h freezing at −20°C and thawing at room temperature. After cycling treatment, the resulting hydrogels with geometries of 90 × 90 × 1 mm were taken out from the molds and used as the final products.

### 4.3 Photo‐controlled polymerization of pyrrole

The PVA/SA‐Na hydrogel hydrogels were immersed in a FeCl_3_ aqueous solution (0.05 M) for 4 h, which were then transferred into a hydrogen chloride aqueous solution (1 M) containing 5 wt% pyrrole. After 3 h the samples were taken out and were further immersed in excessive deionized water for 12 h for purification. To create PPy patterns, the hydrogel samples after immersion in FeCl_3_ aqueous solution were spatially irradiated under UV light (374 mW/cm^2^) for certain times and then immersed in the acidic monomer solution. Home‐made photo‐masks prepared by cutting of aluminum foil papers were used to help the creation of PPy patterns.

### 4.4 Characterizations

Fourier transform infrared spectra (FTIR) were obtained using a Thermo Scientific FTIR spectrometer (PerkingElmer Frontier FTIR) in the range of 4000‐400 cm^−1^ and an accumulation of 16 scans per analysis. XPS measurements were conducted on an AXIS ULTRA multifunctional spectrometer (Kratos Analytical Ltd, Kratos), and the binding energy of C1s was shifted to 284.8 eV as the reference. The microstructures of the hydrogels were investigated by scanning electrical microscopy (SU8220, Hitachi). The viscoelastic properties of the hydrogels were investigated using a rheometer (MCR 302 Rheometer, Anton Paar). The samples (25 mm in diameter and 1 mm in thickness) were placed between two parallel plates of the instrument with a gap of 1 mm. Tensile tests were conducted on a Suns UTM2103 (Shenzhen Sun) universal tensile test machine applying a 1 kN load cell. The sample dimensions are 20.0 × 5.0 × 1.0 mm, and the applied stretching rate is 20 mm/min. The PPy patterns were examined using confocal laser scanning microscopy (Keyence, VK‐X250) with the following settings: a laser wavelength of 408 nm, a power of 0.95 mW, and fully automatic scanning. The electric resistance of the prepared samples was tested using a Shanghai Chenhua CHI660E electrochemical workstation, and the main characterization method was electrochemical impedance spectroscopy.

## CONFLICT OF INTEREST STATEMENT

The authors declare no conflicts of interest.

## ETHICS STATEMENT

No animal or human experiments were involved in this study.

## Supporting information

Supporting Information S1

## Data Availability

The data that support the findings of this study are available from the corresponding author upon reasonable request.
