# Supplementary material for: Designable polypyrrole pattern in hydrogel achieved by photo‐controllable concentration of Fe3+ initiator
Source: Smart Mol. 2024 Jun 27;2(3):e20240015. doi: 10.1002/smo.20240015 (PMC12118226; doi:10.1002/smo.20240015)
Supplement: Supplementary file 1 — Supporting Information S1 [file SMO2-2-e20240015-s001.docx]

Supporting Information

Designable polypyrrole pattern in hydrogel achieved by photo-controllable concentration of Fe^3+^ initiator

Xinyu Zhao^#^, Huidong Xu^#^, Zhao-Tie Liu, Guo Li*, Jinqiang Jiang, and Zhong-Wen Liu*

Key Laboratory of Syngas Conversion of Shaanxi Province, School of Chemistry and Chemical Engineering, Shaanxi Normal University, Xi’an, Shaanxi Province 710062, China.

*: Corresponding authors, E-mail: [liguo@snnu.edu.cn](mailto:liguo@snnu.edu.cn) (G. L.) [zwliu@snnu.edu.cn](mailto:zwliu@snnu.edu.cn) (Z.-W. Liu)


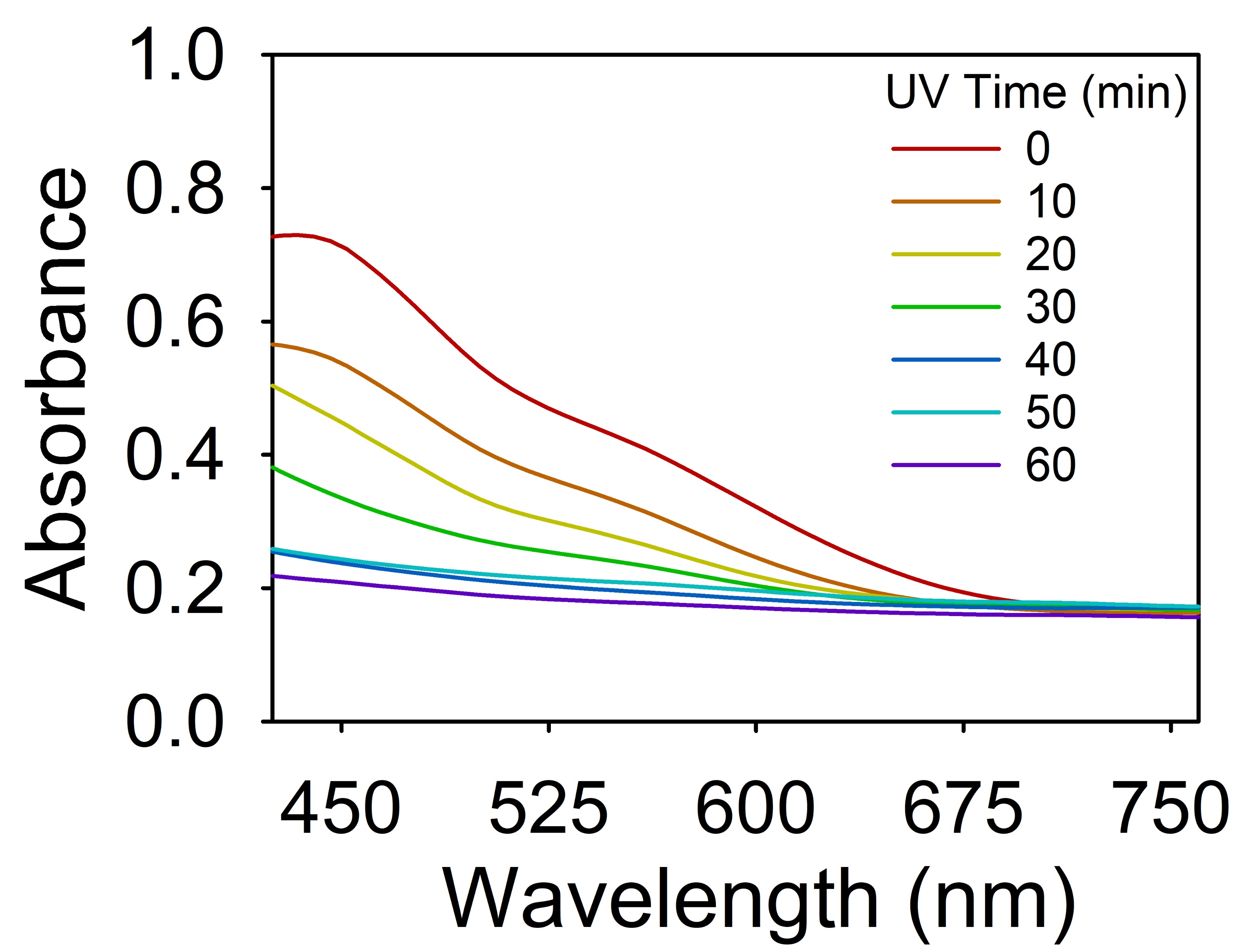


**Figure S1**. UV-Vis spectra of PVA/SA-Fe samples after different times of UV light irradiation.


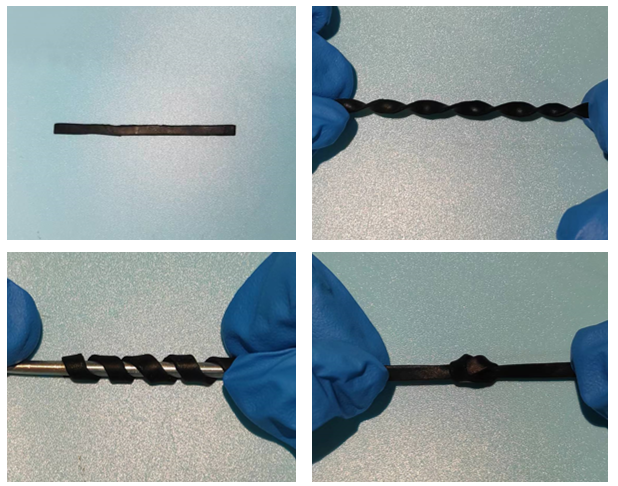


**Figure S2**. Images demonstrating that the developed PVA/SA/PPy-0 samples can tolerate different types of deformation without observable dehiscence.


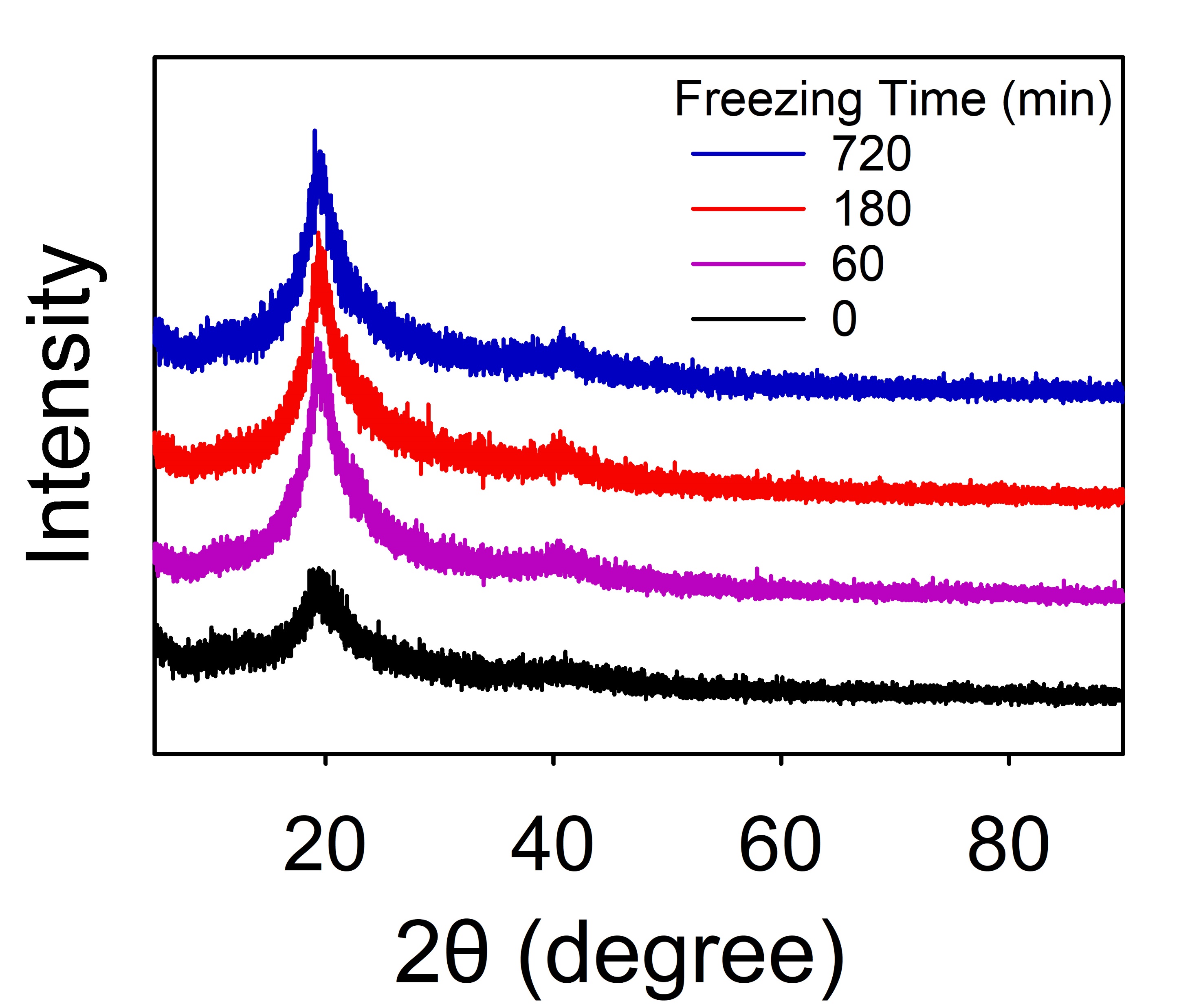


**Figure S3**. XRD curves of PVA/SA-Na samples with different freezing times during freezing/thawing treatment.


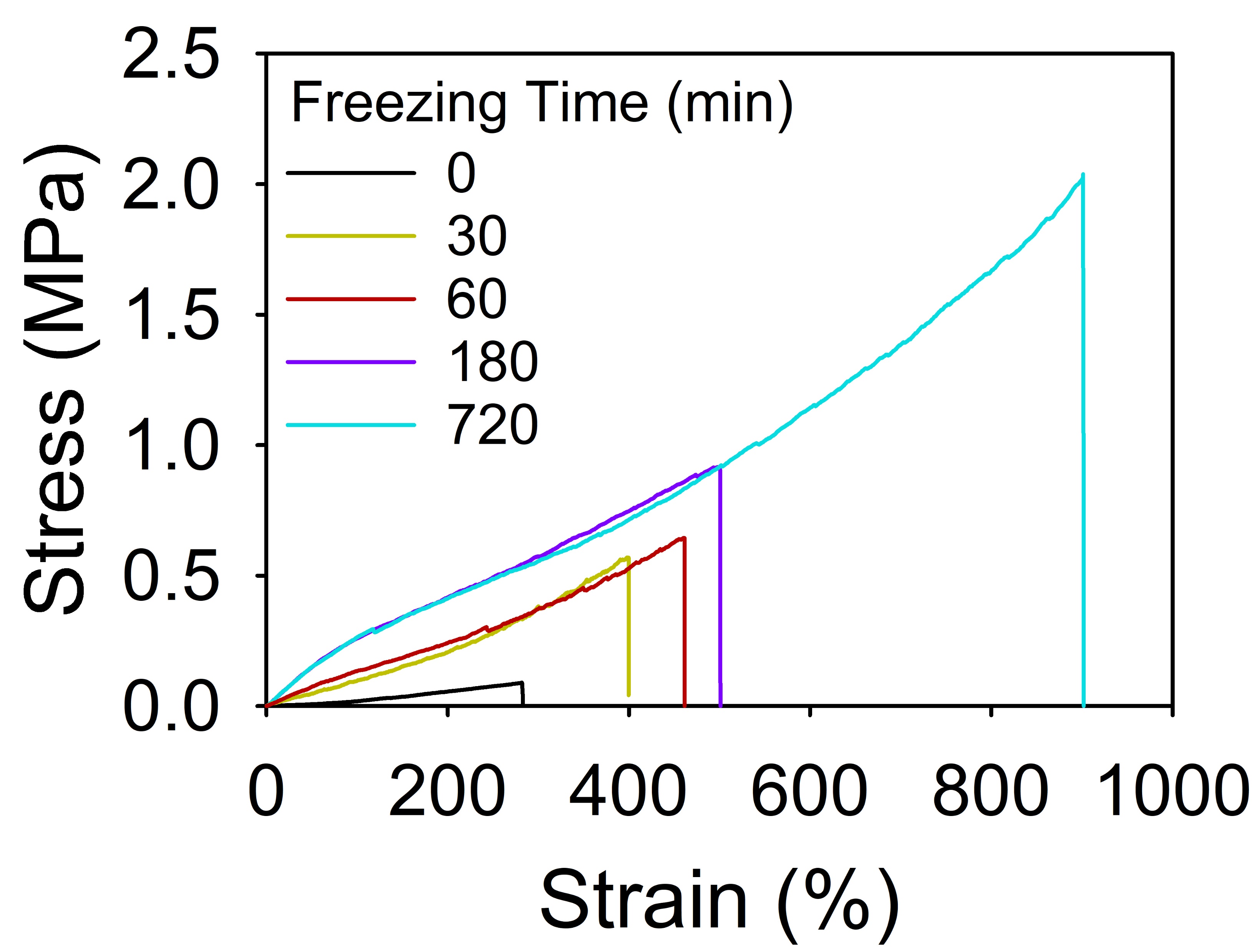


**Figure S4**. Tensile behaviors of PVA/SA-Na samples with different freezing times during freezing/thawing treatment.


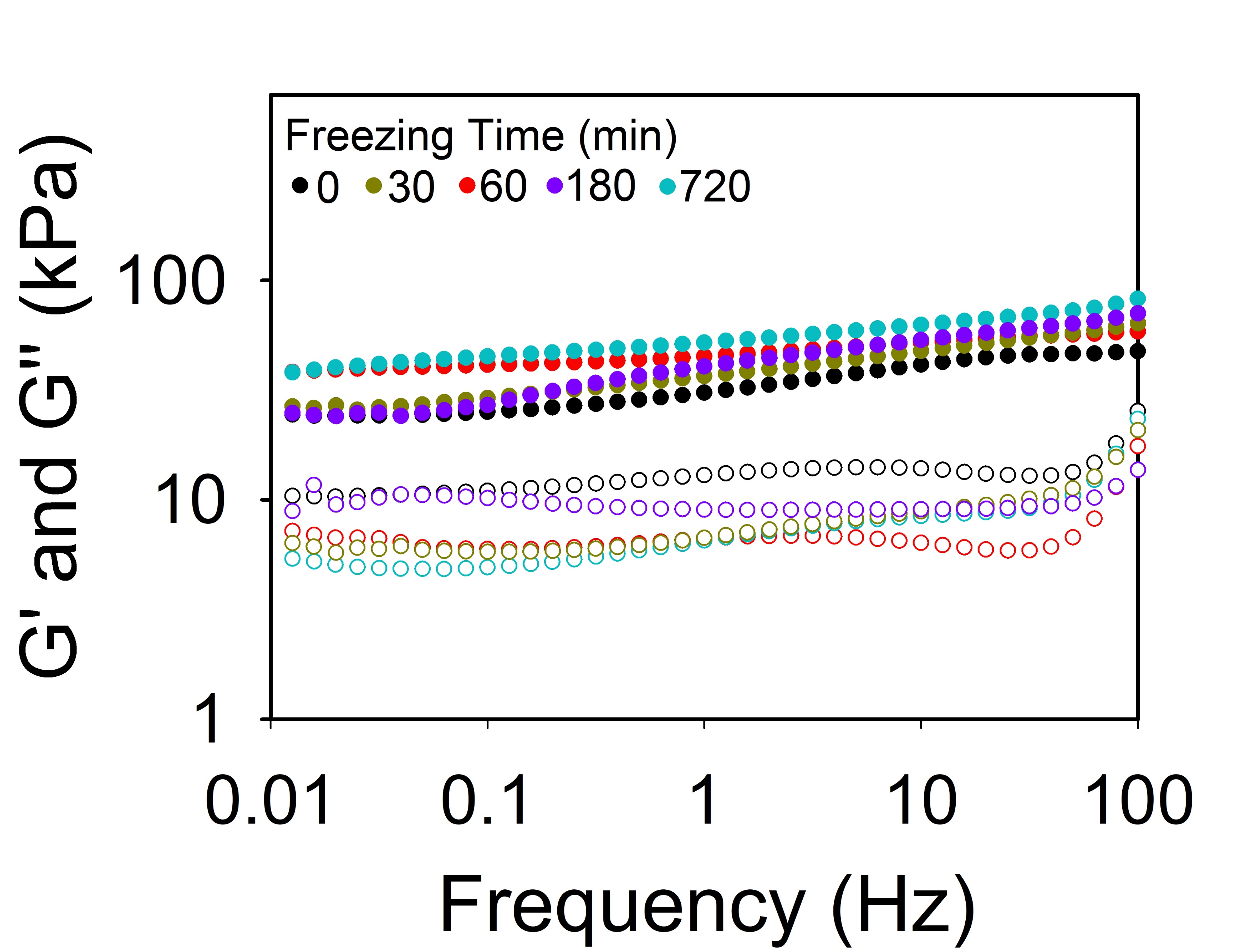


**Figure S5**. Frequency-dependent changes of storage and loss moduli of PVA/SA-Na samples with different freezing times during freezing/thawing treatment.


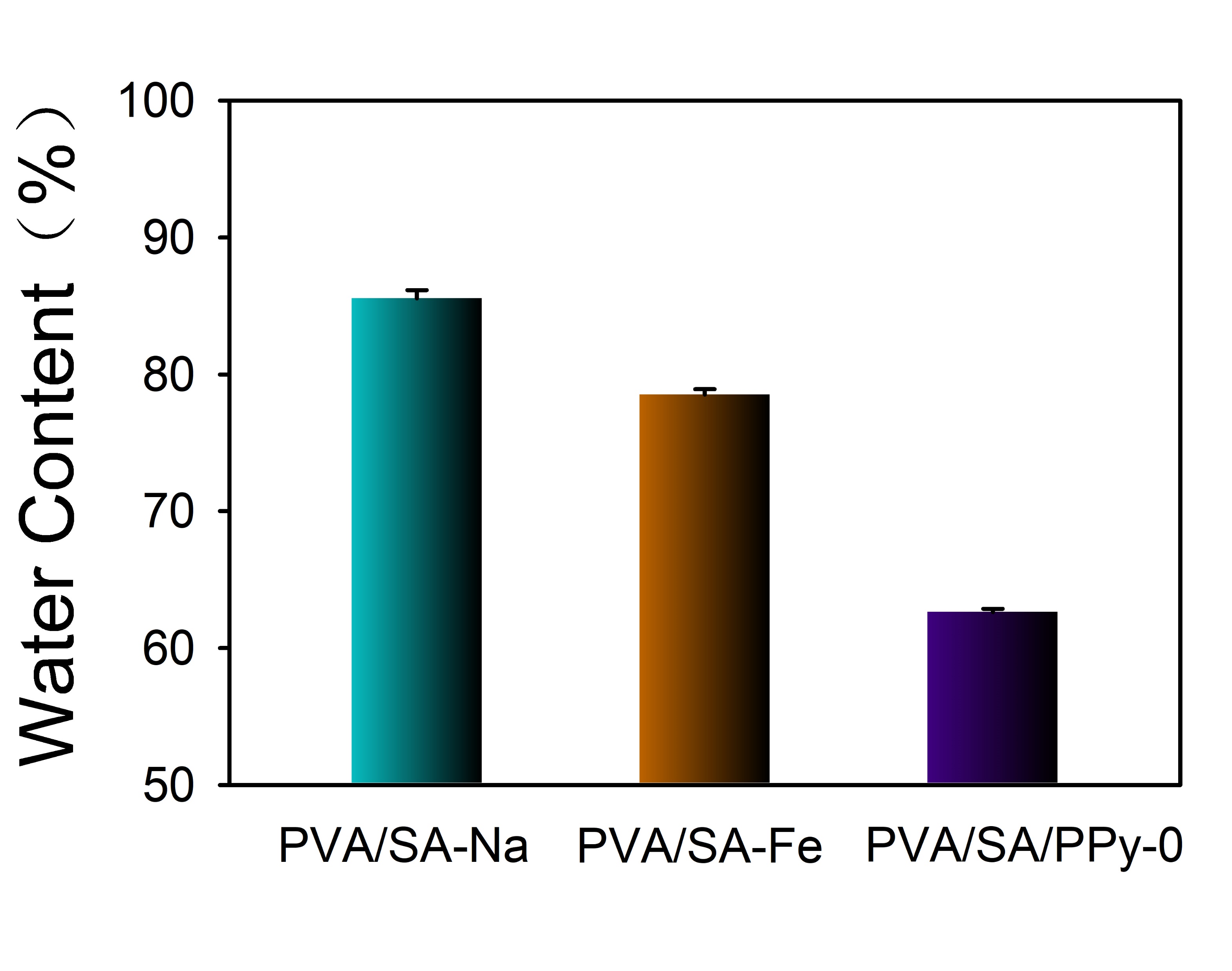


**Figure S6**. Columns showing the equilibrium water contents of PVA/SA-Na, PVA/SA-Fe, and PVA/SA-PPy-0.
